# Extended Phenotyping and Functional Validation Facilitate Diagnosis of a Complex Patient Harboring Genetic Variants in *MCCC1* and *GNB5* Causing Overlapping Phenotypes

**DOI:** 10.3390/genes12091352

**Published:** 2021-08-29

**Authors:** Zhuo Shao, Ikuo Masuho, Anupreet Tumber, Jason T. Maynes, Erika Tavares, Asim Ali, Stacy Hewson, Andreas Schulze, Peter Kannu, Kirill A. Martemyanov, Ajoy Vincent

**Affiliations:** 1 Division of Clinical and Metabolic Genetics, Hospital for Sick Children, Toronto, ON M5G 1X8, Canada; shawn.shao@sickkids.ca (Z.S.); stacy.hewson@sickkids.ca (S.H.); andreas.schulze@sickkids.ca (A.S.); 2 Department of Neuroscience, The Scripps Research Institute, 130 Scripps Way, Jupiter, FL 33458, USA; imasuho@scripps.edu (I.M.); Kirill@scripps.edu (K.A.M.); 3 Pediatrics and Rare Diseases Group, Sanford Research, 2301 East 60th Street North, Sioux Falls, SD 57104, USA; 4 Department of Ophthalmology and Vision Sciences, Hospital for Sick Children, Toronto, ON M5G 1X8, Canada; anupreet.tumber@sickkids.ca (A.T.); asim.ali@sickkids.ca (A.A.); 5 Division of Molecular Medicine, Hospital for Sick Children, Toronto, ON M5G 1X8, Canada; jason.maynes@sickkids.ca; 6 Department of Anesthesiology, Faculty of Medicine, University of Toronto, Toronto, ON M5S 1A8, Canada; 7 Genetics and Genome Biology, Hospital for Sick Children, Toronto, ON M5G 1X8, Canada; erika.tavares@sickkids.ca; 8 Department of Ophthalmology and Vision Sciences, University of Toronto, Toronto, ON M5S 1A8, Canada; 9 Department of Paediatrics, University of Toronto, Toronto, ON M5S 1A1, Canada; 10 Department of Biochemistry, University of Toronto, Toronto, ON M5S 1A8, Canada; 11 Medical Genetics, University of Alberta, Edmonton, AB T6G 2R3, Canada; kannu@ualberta.ca

**Keywords:** guanine nucleotide binding protein (G-protein), beta5, human, receptors, G-protein-coupled, electroretinography, metabolism, inborn errors, whole exome sequencing, developmental disabilities, GNB5, MCCC1

## Abstract

Identifying multiple ultra-rare genetic syndromes with overlapping phenotypes is a diagnostic conundrum in clinical genetics. This study investigated the pathogenicity of a homozygous missense variant in *GNB5* (*GNB5L;* NM_016194.4: c.920T > G (p. Leu307Arg); *GNB5S;* NM_006578.4: c.794T > G (p. Leu265Arg)) identified through exome sequencing in a female child who also had 3-methylcrotonyl-CoA carboxylase (3-MCC) deficiency (newborn screening positive) and hemoglobin E trait. The proband presented with early-onset intellectual disability, the severity of which was more in keeping with *GNB5*-related disorder than 3-MCC deficiency. She later developed bradycardia and cardiac arrest, and upon re-phenotyping showed cone photo-transduction recovery deficit, all known only to *GNB5*-related disorders. Patient-derived fibroblast assays showed preserved GNB5S expression, but bioluminescence resonance energy transfer assay showed abolished function of the variant reconstituted Gβ5S containing RGS complexes for deactivation of D2 dopamine receptor activity, confirming variant pathogenicity. This study highlights the need for precise phenotyping and functional assays to facilitate variant classification and clinical diagnosis in patients with complex medical conditions.

## 1. Introduction

Whole exome sequencing (WES) is increasingly used for the diagnosis of patients with suspected Mendelian genetic disorders [1]. The diagnostic yield of WES has significantly improved due to the rapid accumulation of knowledge in genomic medicine, new discoveries of disease-causing genes, and expanded understanding of the phenotypic spectrum of known syndromes [2]. However, interpretation of the pathogenicity of missense variants remains challenging, especially in ultra-rare conditions where the phenotypic spectrum is not yet fully understood [3]. An added layer of complexity occurs when variants are identified in multiple genes that lead to syndromes presenting with overlapping phenotypes [4].

Biallelic pathogenic variants in the G protein subunit beta 5 (*GNB5*) gene are associated with a broad range of clinical presentations, with fewer than 30 patients reported in the literature [5,6,7,8,9,10,11]. Homozygous or compound heterozygous variants with at least one null allele in *GNB5* cause intellectual developmental disorder with cardiac arrhythmia (IDDCA; MIM: 617173) associated with severe intellectual disability (ID) [12]. Biallelic missense variants in *GNB5* cause language delay and attention deficit-hyperactivity disorder/cognitive impairment with or without cardiac arrhythmia (LADCI; MIM: 617182), [12,13] associated with mild or no ID. Cardiac arrythmia due to sick sinus syndrome is a salient feature of both IDDCA and LADCI, with some patients requiring pacemaker implantation [12,13]. Hypotonia and seizures typically occur in patients with IDDCA [14], and a retinal signaling defect featuring bradyopsia and rod ON-bipolar dysfunction is only described in IDDCA [15]. However, a recent study reports certain biallelic missense variants to result in loss of Gβ5 protein function leading to phenotypes more in keeping with IDDCA [16]. The biological mechanisms of how impaired Gβ5 function causes IDDCA or LADCI is not fully understood. Efforts were made to generate induced human pluripotent stem cell lines from patients with *GNB5*-related disorders in order to study cell type-specific impact of Gβ5 dysfunction [17]. The evidence from *Gnb5*-knockout models (zebrafish and mice) demonstrated neuronal and cardiac phenotypes observed in IDDCA and LADCI patients [12,18,19,20,21].

*GNB5* encodes the Gβ5 protein, an integral part of the G-protein coupled receptor (GPCR) machinery. GPCRs activate heterotrimeric G proteins by catalyzing the exchange of GDP to GTP on the Gα subunit, which results in its dissociation from the Gβγ dimer. This cascade is deactivated by the regulator of G-protein signaling (RGS) proteins, which accelerate the GTP hydrolysis on the Gα subunits back to a GDP [22]. Gβ5, unlike other Gβ subunits, does not form functional trimeric G proteins [23,24]. Instead, Gβ5 interacts with the R7 subfamily of RGS proteins (including RGS9) and stabilizes them, which potentiates R7 RGS proteins’ role in the deactivation of GPCR signaling [22,25,26,27]. There are two splice isoforms of *G*β5: the Gβ5-long (Gβ5L) form contains 42 extra amino acid residues at the N-terminus compared to the Gβ5-short (Gβ5S) form [28]. Both isoforms contain the seven WD40 regions. Gβ5S is highly expressed throughout the central nervous system (CNS) [29,30] and broadly expressed across peripheral tissues including cardiac myocytes and macrophages [31,32]. In the retina, Gβ5S is localized to the retinal ON-bipolar cells [33]. In contrast, Gβ5L expression in the body is limited to the photoreceptor outer segments in the retina [34].

3-Methylcrotonyl-CoA carboxylase (3-MCC) is an enzyme involved in catabolism of leucine, isoprenoids, and mevalonate shunt pathways. Like other carboxylases, it is a biotin-containing ATP-dependent enzyme. Isolated 3-MCC deficiency leads to 3-methylcrotonylglycinuria, which was previously reported (1970s to early 2000s) to cause vomiting, feeding difficulty, failure to thrive, hypotonia, seizure, and mild ID (MIM 210200). Recent long-term follow-up studies of patients with 3-MCC deficiency identified through newborn screening revealed this condition to have low clinical expressivity and penetrance [35]; the majority (90%) are asymptomatic, and fewer than 10% have mild neurological symptoms, which may be unrelated to the condition [36]. As a result, newborn screening for 3-MCC deficiency was removed in some jurisdictions in North America [37,38].

This is a case report of a female diagnosed with 3-MCC deficiency following positive newborn screening and was later diagnosed with hemoglobin E trait. Since her phenotypic features deviated from the clinical presentation described in isolated 3-MCC deficiency, WES was arranged to investigate for any additional genetic etiology. A homozygous missense variant of uncertain significance was identified in *GNB5* (*GNB5L;* NM_016194.4: c.920T > G (p. Leu307Arg); *GNB5S;* NM_006578.4: c.794T > G (p. Leu265Arg)). To delineate the contribution of this *GNB5* variant to the proband’s phenotype, we performed detailed clinical re-phenotyping and functional assays. Comparing the proband’s clinical findings with the known phenotypic spectrum of 3-MCC and *GNB5*-related disorders revealed that the cardiac and ocular features are unique to *GNB5*- disorders. The variant pathogenicity was confirmed using bioluminescence resonance energy transfer (BRET) assay [39,40].

## 2. Materials and Methods

The study was approved by the Research Ethics Board at the Hospital for Sick Children Toronto (REB #1000017804), and written informed consent was obtained from the parent; the study protocols adhered to the tenets of the Declaration of Helsinki. Clinical information was ascertained by chart review of laboratory reports; neuroimaging; and electrophysiology studies including electroencephalogram (EEG), electrocardiogram (ECG), and electroretinogram (ERG), as well as genetic testing results. Clinical notes and neuropsychiatric evaluation were used to establish the developmental progress, hearing, vision, and physical examination assessments.

### 2.1. Genetic Testing 

Single Genetic testing for *MCCC1* was performed (2009) at a CLIA certified laboratory (GeneDx, Gaithersburg, MD, USA), which used PCR-based DNA amplification and bidirectional sequencing of all exons. Clinical standard whole exome sequencing (WES) was carried out by the same lab (2017) for the proband and her parents (Trio-WES). Exons were captured and sequenced using massively parallel sequencing technique from DNA obtained from blood. Each individual’s sequence was then compared to reference sequences, other individuals’ sequences from the family, and from control individuals; subsequently, phenotype-driven gene lists were generated using the Human Gene Mutation Database (http://www.hgmd.cf.ac.uk/ac/index.php) and Human Phenotype Ontology (https://hpo.jax.org/app/) genotype–phenotype associations. Additional resources such as GnomAD NHLBI Exome Sequencing Project [41], OMIM (https://www.omim.org/), PubMed, and Clinvar were used to evaluate genes and detect sequence changes of interest, which were then interpreted according to the American College of Medical Genetics and Genomics’ (ACMG) standards and guidelines for sequence variants interpretation [42].

### 2.2. Electroretinogram

An extended version of the ISCEV standard full-field ERG was performed, as previously described [15]. Scotopic ERG stimulus intensities ranged from 0.001 to 30 cd.s.m^−2^; the standard (DA 3.0), strong (DA 10.0), and maximal (DA 30.0) flashes were tested at standard and extended inter stimulus intervals, up to 60s. Photopic ERG stimulus intensities ranged from 3.0 to 30 cd.s.m^−2^ and were tested with a background luminance of 30 cd.m^−2^ [15,43,44]. A standard 30Hz flicker ERG was performed for 0.5 s, 1 s, 3 s, and 6 s durations.

### 2.3. Fibroblast Cell Culture

Fibroblast cell line was derived from patient′s skin biopsy. Three passage (P3) fibroblast was harvested after confluence and stored in liquid nitrogen. For gene expression and protein localization studies, previously stored fibroblast cell line was thawed and plated in 10 mm and 15 mm tissue culture plates. The minimum essential medium Eagle–alpha medium (AMEM; Gibco™ Cat#: 12571063) with 10% fetal bovine serum (FBS) was changed daily, and cells were cultured at 37 °C under a 5% CO_2_ at atmospheric pressure.

### 2.4. Digital PCR

The fibroblast cell line was harvested after trypsinization, and mRNA was extracted using RNeasy^®^ kit (Qiagen, Cat. 74104) following the manufacture’s protocol. Total RNA (1–5 µg per sample) was diluted into a volume of 15 µL and incubated at 70 °C for 10 min to dissociate the secondary structure. A master mix of 6 µL of 5× first strand buffer, 3 µL of DTT, 2 µL of dNTP, 1 µL of RNase, and 1 µL of oligo-dT with 2 µL of M-MLV reverse transcription enzyme was added to the diluted RNA sample and incubated at 42 °C for 60 min followed by 5 min of inactivation at 94 °C. These cDNA samples were then used for either digital PCR or regular PCR. The digital PCR was carried out by the Hospital for Sick Children Research Center Genetic Analysis Facility. Primer sequences for regular PCRs were forward: (5′ to 3′): CCCTCAGAAACTGGAAACACCT and reverse (5′ to 3′): CCAAACAGGATGGAGACCCG. Digital PCRs were carried out using commercially available dPCR primer set (Thermo Fisher Scientific, Waltham, MA, USA, Cat # Hs00275095_m1). As sequence homology of the two isoforms in this region is 100%, these primer pairs will capture both isoforms; however, since the long form is exclusively expressed in photoreceptors, amplification from cDNA generated from fibroblast cells should be exclusively GNB5S.

### 2.5. Cultures of HEK293T/17 Cells

HEK293T/17 cells were obtained from ATTC (Manassas, VA, USA) and grown in DMEM supplemented with 10% FBS, minimum Eagle’s medium non-essential amino acids, 1 mM sodium pyruvate, and antibiotics (100 units/mL penicillin and 100 mg/mL streptomycin) at 37 °C in a humidified incubator containing 5% CO_2_.

### 2.6. cDNA Constructs

GαoA (GenBank: NM_020988), RGS9-2, and Gβ5S (GenBank: NM_006578) in pcDNA3.1(+) were purchased from cDNA Resource Center (https://www.cdna.org Accessed on 26 August 2021). Flag-tagged dopamine D2 receptors (GenBank: NM_000795) containing the hemagglutinin signal sequence (KTIIALSYIFCLVFA) at the N terminus was a gift from Dr. Abraham Kovoor. Venus 156-239-Gβ1 (amino acids 156-239 of Venus fused to a GGSGGG linker at the N terminus of Gβ1 without the first methionine (GenBank: NM_002074)) and Venus 1-155-Gγ2 (amino acids 1-155 of Venus fused to a GGSGGG linker at the N terminus of Gγ2 (GenBank: NM_053064)) were gifts from Dr. Nevin A. Lambert [45]. The masGRK3ct-Nluc-HA construct was constructed by introducing HA tag at the C terminus of masGRK3ct-Nluc reported previously [46]. The R7BP construct was reported previously [47].

### 2.7. Transfection

We coated 3.5 cm culture dishes during incubation for 5 min at 37 °C with 1 mL of Matrigel solution (approximately 10 mg/mL growth factor-reduced Matrigel in culture medium). Cells were seeded into the 3.5 cm dishes containing Matrigel solution at a density of 2 × 10^6^ cells/dish. After 2 h, expression constructs (total 5 mg/dish) were transfected into the cells using PLUS (5 ml/dish) and Lipofectamine LTX (6 ml/dish) reagents. Dopamine D2 receptor (D2R) (1), GαoA (2), Venus 156-239-Gβ1 (1), Venus 1-155-Gγ2 (1), masGRK3ct-Nluc-HA (1), RGS9-2 (0.5), and Gβ5S (0.5) were transfected (the number in parentheses indicates the ratio of transfected DNA (ratio 1 = 0.21 mg)). An empty vector (pcDNA3.1(+)) was used to normalize the amount of transfected DNA.

### 2.8. Bioluminescence Resonance Energy Transfer (BRET)

Cellular measurements of BRET between Venus-Gβ1γ2 and masGRK3ct-Nluc-HA were performed to examine GAP activity of RGS9-2/Gβ5s complex in living cells [40,48,49]. Then, at 16 to 24 h post-transfection, HEK293T/17 cells were washed once with BRET buffer (Dulbecco’s phosphate-buffered saline (PBS) containing 0.5 mM MgCl_2_ and 0.1% glucose) and detached by gentle pipetting over the monolayer. Cells were harvested by centrifugation at 500× *g* for 5 min and resuspended in BRET buffer. Approximately 50,000 to 100,000 cells per well were distributed in 96-well flatbottomed white microplates (Greiner Bio-One, Kremsmunster, Austria). The NanoLuc (Nluc) substrate, furimazine [50], was purchased from Promega and used according to the manufacturer’s instructions. BRET measurements were made using a microplate reader (POLARstar Omega; BMG Labtech, Ortenberg, Germany) equipped with two emission photomultiplier tubes, allowing us to detect two emissions simultaneously with the highest possible resolution of 20 ms per data point. All measurements were performed at room temperature. To activate and then deactivate, we applied the final concentration of 100 μM dopamine and 100 μM haloperidol on the transfected cells to control the activity of those GPCRs. The BRET signal was determined by calculating the ratio of the light emitted by the Venus- Gβ1γ2 (535 nm with a 30 nm band path width) over the light emitted by the masGRK3ct-Nluc-HA (475 nm with a 30 nm band path width). The average lowest value (basal BRET ratio) recorded after haloperidol application was subtracted from the experimental BRET signal values, and the resulting difference (DBRET ratio) was normalized against the maximal DBRET value recorded upon agonist stimulation. The rate constants (1/μ) of the deactivation phases were obtained by fitting a single exponential curve to the traces with Clampfit 10.3. *k*_GAP_ rate constants were determined by subtracting the basal deactivation rate (*k*_app_) from the deactivation rate measured in the presence of exogenous RGS protein. Obtained *k*_GAP_ rate constants were used to quantify GAP activity. Bioluminescence resonance energy transfer (BRET)-based signaling assay and measuring the GAP of Regulator G-protein signaling (RGS) complexes in expression assays were used to investigate the impact of the variant on the function of Gβ5S.

### 2.9. Western Blot

A portion of the cells collected for the BRET assay was used for Western blotting. Protein extracts (40 μg) from the transfected HEK293T/17 cells were loaded to commercially made 4–12% Bolt gel, separated by voltage potential using Mini Gel Tank (Thermofisher, Waltham, MA, USA), and transferred to polyscreen polyvinylidene difluoride transfer membrane (PerkinElmer, Waltham, MA, USA) using iBlot™ 2 Gel Transfer Device (Thermo Fisher Scientific, Waltham, MA, USA). Ponceau stain was used to demonstrate the equal loading of proteins in each well. Respective proteins were revealed by anti-RGS9 antibody (Proteintech, Rosemont, IL, USA, 17970-1-AP), anti-Gβ5 ATDG antibody [51], anti-R7BP antibody [29], and anti-GAPDH antibody (Millipore, Burlington, MA, USA, MAB374). Anti-Gβ5 antibody and anti-R7BP antibody were gifts from Dr. William Simonds (NIDDK, National Institutes of Health, Bethesda, MD, USA).

### 2.10. Statistical Analysis

Data are presented as mean ± SEM of three biological replicates using independent transfection. Comparisons between groups were made with either two-tail unpaired Student’s *t*-test or analysis of variance (ANOVA), followed by post hoc Bonferroni correction or Tukey multiple-comparison for comparison among means. *p* < 0.05 was considered statistically significant. (* *p* < 0.05; ** *p* < 0.01; *** *p* < 0.001; **** *p* < 0.0001).

## 3. Results

### 3.1. General Phenotype of the Proband

**Figure 1:**
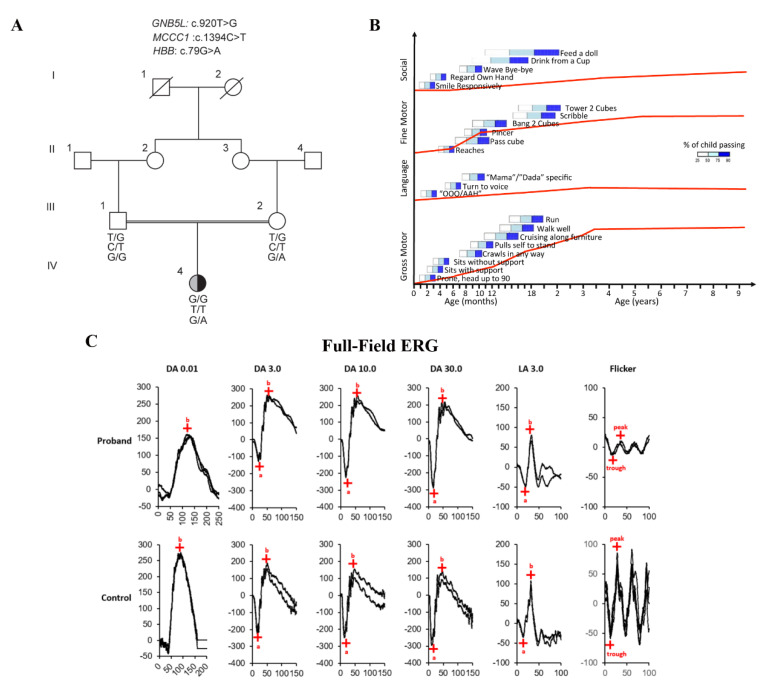

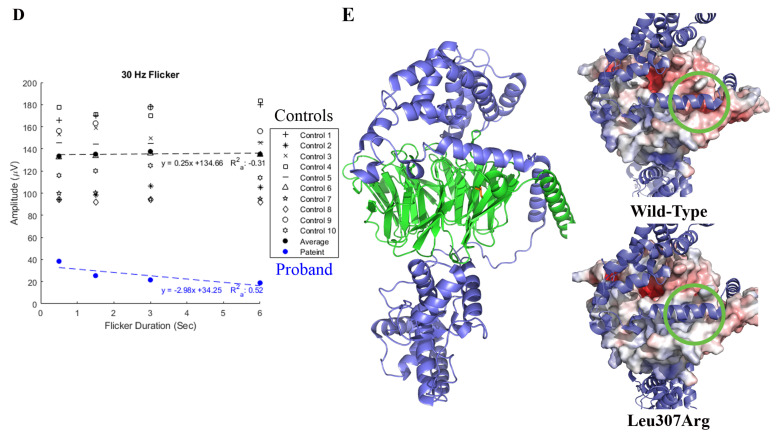
Clinical phenotype and homology modeling. (**A**) Family pedigree and segregation results. (**B**) The developmental milestones plotted on a modified Denver II chart demonstrated severe developmental delay across all domains. (**C**) Electroretinogram (ERG) results: Compared to the control, all scotopic responses (DA 0.01, DA 3.0, and DA 10.0) and the single flash photopic responses (LA 3.0) were normal; the flicker ERG amplitudes were severely reduced in excess of high myopia. (**D**) Progressive deficit in cone photo-transduction recovery was elicited using a 30 Hz ERG flicker protocol tested over multiple stimulus durations (0.5 s, 1.5 s, 3.0 s, 6.0 s). Ten healthy individuals were recruited to establish the normal range; controls showed stable amplitudes regardless of the stimulus duration, whereas the proband showed a progressive decrease in flicker amplitudes with increasing stimulus duration. (**E**) X-ray crystal structure of the complex between Gβ5 (green) and the GAP protein RGS9 (blue). The GTPase modifier RGS9 interacted with the two main β-propeller surfaces of Gβ5. On the superior surface of the propeller, a helix of RGS9 (residues 239–255) bound into a Gβ5 groove containing Leu307 (shown in orange sticks) (left). The electrostatic potential of Gβ5 is illustrated on the right with the wild type on top and modeled Leu307Arg on the bottom (colored as tradition, with red for electronegative, blue for electropositive, and white for electroneutral). Although the overall architecture of the surface was not predicted to be grossly altered, the mutation induced a significant shift in the electrostatic surface potential (indicated in the green circle). The change in potential demonstrated how Leu307Arg affects the ability of partners to bind, reducing the affinity of the predominantly hydrophobic helix of RGS9 and affecting complex formation and quaternary structure. Figure made in Pymol (The PyMOL Molecular Graphics System, Version 1.8.6.2 Schrödinger, LLC).

The female proband (currently 11 years of age) born to consanguineous parents of Cambodian descent (Figure 1A) has been followed in the inherited metabolic disease clinic since birth due to 3-MCC deficiency identified on newborn screening. Further testing in the fibroblasts showed severely reduced 3-methylcrotonyl-CoA carboxylase activity (0.5pmol/min/mg protein; normal: >31), and normal propionyl-CoA carboxylase (105pmol/min/mg protein; normal: >70) and pyruvate carboxylase (15 pmol/min/mg protein; normal: >6) activity (University of California, San Diego, CA, USA). Increased urinary levels of 3-hydroxyisovaleric acid and 3-methylcrotonylglycine were detected. These results were consistent with isolated 3-MCC deficiency, and a homozygous likely pathogenic variant was identified in *MCCC1* gene (NM_020166.5 c.1394C > T (p. Thr465Ile); as per ACMG variant interpretation guideline [42], the variant is classified as likely pathogenic: PS3, PM2, PP2, PP3). She was treated with biotin and L-carnitine since 4 weeks of age. She was found to have severe developmental delay at 6 months of age. Brain magnetic resonance imaging and spectroscopy performed at 11 months were unremarkable. At 1 year of age, an abdominal ultrasound showed mild splenomegaly at 9cm (normal < 7cm) [52] with normal liver size. Hematological evaluation showed no anemia, but the child was diagnosed with hemoglobin E trait following identification of a known heterozygous pathogenic variant in *HBB* [(NM_000518.4 c.79G > A (p. Glu27Lys); (ClinVar: RCV000778330.1)], which explained the splenomegaly. She continued to exhibit profound developmental delay; hence, chromosomal microarray analysis was performed at 28 months of age, which showed normal chromosome dosage. At 2 years 7 months, she had one episode of suspected seizure and brief asystole during a febrile illness. An electroencephalogram demonstrated non-specific generalized diffused slow background in keeping with mild diffuse encephalopathy but showed no seizure activity. Electrocardiogram monitoring showed sinus bradycardia (40 beats per minute) while sleeping. She had one additional episode of asystole at 6.5 years during anesthesia. A formal developmental assessment at 3.5 years showed global developmental delay with gross motor skills equivalent to an 11-month-old, fine motor skills equivalent to a 10-month-old, social skills equivalent to a 7-month-old, and language equivalent to a 4-month-old. Minimal new skills were attained afterwards consistent with severe ID (Figure 1B). Since the severity of her ID as well as her cardiac rhythm abnormalities were unexplained by 3-MCC deficiency, WES was arranged at 7 years 10 months.

### 3.2. Molecular Genetic Result

The WES revealed a homozygous missense variant of uncertain significance in *GNB5* annotated as c.920T > G (p. Leu307Arg) [*GNB5L;* NM_016194.4] and c.794T > G (p. Leu265Arg), [*GNB5S*; NM_006578.4]; parents were both heterozygous for the variant. The variant has never been previously reported or observed in large population cohorts such as the Genome Aggregation Database [41]. This nucleotide is considered highly conserved (phyloP: 8.94 [−20.0;10.0]), and the amino acid leucine at position 307 (*GNB5L*) is evolutionarily conserved in vertebrates including zebrafish. This variant causes a non-conservative amino acid substitution, as leucine and arginine residues differ in polarity, charge, and size (Grantham score 102 (0-215); moderately radical). In silico analysis predicted this variant to be probably damaging [SIFT (v6.2.0): Deleterious (score: 0) [53]; MutationTaster (v2013): disease causing (prob: 1) [54], PolyPhen-2: probably damaging, (score: 0.993) [55]].

### 3.3. Ocular Phenotype

Her ocular features included progressive myopia, first identified at 2 years of age. At 7 years, she responded to light and familiar faces, but had high myopia (−16.50D and −15.50D in the right and left eyes, respectively). Her retina showed changes expected in myopia that included tilted optic disc, peripapillary atrophy, and tessellated fundus. Given her severe ID, wearing spectacles or contact lens correction was not possible and, hence, phakic intraocular lens implantation was performed at 8 years of age. Following WES results, retinal re-phenotyping was performed using standard and extended protocol electroretinograms (ERGs) [15,44,56]. The ERG revealed normal rod (scotopic) responses; the cone (photopic) responses were normal to a slow flash (2 Hz) but demonstrated severely reduced amplitudes to a fast flicker stimulus (30 Hz flicker, 3 s duration) in excess of what is expected for her myopia (Figure 1C). These results suggested normal rod photo-transduction recovery and rod bipolar cell signaling; however, cone photo-transduction recovery was deemed affected. Cone phototransduction recovery deficit was confirmed using a novel 30 Hz flicker protocol incorporating various stimulus durations (0.5, 1.5, 3, and 6 s). The proband’s flicker amplitudes progressively reduced with increasing stimulus duration, whereas controls had stable flicker amplitudes regardless of the stimulus duration (Figure 1D). 

The proband′s phenotype was compared to that of 3-MCC, HbE trait, and *GNB5*-related disorders (Table 1). Overall, the cardiac and retinal features were only known to *GNB5*-related disorder, whereas the neurological and developmental features overlapped between 3-MCC deficiency and *GNB5* disorder, but the severity was out of keeping with 3-MCC deficiency. The mild splenomegaly was consistent with HbE trait.

### 3.4. Proband’s Fibroblast GNB5 Expression

**Figure 2:**
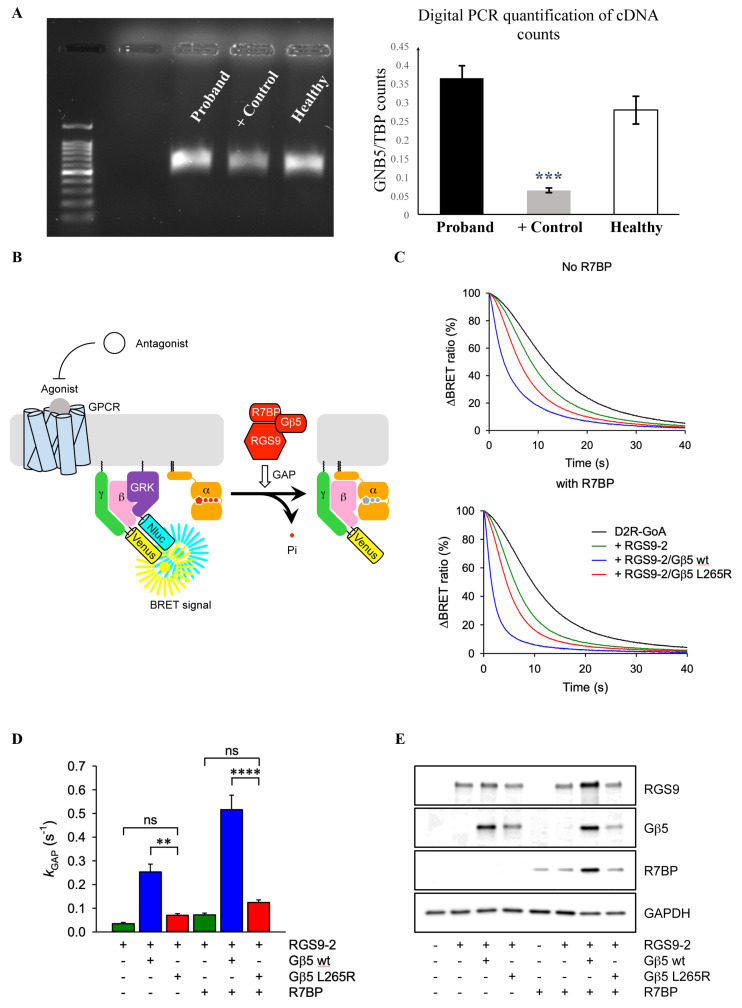
*GNB5* expression and characterization of Gβ5S Leu265Arg mutant in a living cell environment. (**A**) The GNB5S mRNA level was reduced in fibroblasts derived from positive control, but unchanged in the proband with missense variant comparing to healthy control (healthy). The image on the left demonstrates the band of amplified PCR product, and the histogram on the right shows the quantification from digital PCR reactions (*n* = 3; *** *p* < 0.001). (**B**) Schematic of the BRET assay. Agonist-bound GPCR led to the dissociation of inactive heterotrimeric G proteins into active GTP-bound Gα and Venus-Gβγ subunits. The free Venus-Gβγ interacted with the Gβγ-effector mimetic masGRK3ct-Nluc-HA and increased the BRET signal. The application of the antagonist initiated the deactivation of G proteins and decreased the BRET signal. (**C**) Real-time monitoring of G protein deactivation. HEK293T/17 cells were transfected with D2R, GαoA, Venus-Gβγ, and masGRK3ct-Nluc-HA together with RGS9-1/Gβ5s complex. The experiments were performed without or with R7BP. After activation of G protein by 100 μM dopamine, 100 μM haloperidol was applied at time zero, and the BRET signal was followed across time (** *p* < 0.01, **** *p* < 0.0001). κGAP rate constants were determined by subtracting the basal deactivation rate (black line) from the deactivation rate measured in the presence of exogenous RGS protein. The results indicate severe loss in the ability of mutant Gβ5 to accelerate GPCR deactivation kinetics of HEK293T/17-transfected cells. (**D**,**E**) Comparison of GAP activity of RGS9-2 complexes with wild-type and mutant Gβ5S in the presence and absence of R7BP. The impact of L265R mutation on the stability of RGS9-2 complexes was demonstrated by Western blot.

Reverse transcriptase (RT)-PCR and digital PCR on fibroblast cell lines from the proband, positive control [*GNB5S*; c.906C > A(p. Tyr302*) [15]], and a negative control (healthy individual) demonstrated no significant changes in GNB5S mRNA levels in the proband compared to control (Figure 2A). The RT-PCR product was sequenced in the proband and positive control to confirm the product and verify the variants in *GNB5S*.

### 3.5. BRET-Based Gβ5 Functional Analysis

Since the missense variant did not compromise GNB5S expression at transcriptional level in the proband, we examined the impact of the alteration on Gβ5S protein function. The function of Gβ5S was assessed by its ability to support GTPase activating protein (GAP) complex involving brain-specific RGS9-2 and R7BP subunits (Figure 2B). We used a BRET-based strategy to monitor GAP complex activity by measuring the kinetics of G protein (Gαo) deactivation in the reconstituted system containing D2 dopamine receptor (D2R) (Figure 2B). Since Gβ5L is a photoreceptor-specific isoform, we performed functional analysis with more broadly expressed Gβ5S. Inclusion of RGS9-2 with wild-type Gβ5S substantially accelerated termination of D2R response consistent with the GAP activity of the complex (Figure 2C,D). In contrast, when the complex was reconstituted with Leu265Arg mutant of Gβ5S, no such acceleration was observed (Figure 2C,D). Similar results were observed when the complex was additionally reconstituted with R7BP subunit, which further potentiated the activity of the GAP complex with wild-type but not Leu265Arg Gβ5S mutant (Figure 2C,D). Thus, Leu265Arg dramatically reduced the function of RGS9-2 to about 24% and 28% in the presence and absence of R7BP, respectively. To understand the mechanism by which Leu265Arg substitution compromises the activity of the GAP complex, we analyzed the effect of Gβ5S on stabilization of RGS9-2 in the transfected system because Gβ5S is necessary for the expression of R7 RGS proteins in vivo [26]. Western blotting analysis revealed that while wild-type Gβ5S significantly increased the expression of RGS9-2 and augmented the levels of R7BP when present, the Leu265Arg Gβ5S mutant completely failed to do so (Figure 2E). Further, the Leu265Arg mutant of Gβ5S was expressed at lower levels than wild-type Gβ5S. Therefore, we concluded that Leu265Arg substitution compromised the stability of Gβ5S at the protein level and prevented it from supporting the GAP complex.

### 3.6. Gβ5 Crystal Structure Analysis

Consistent with the role of Gβ5L in a large signaling complex, the protein is composed of seven WD40 regions (residues 53 to 340), assembling into a canonical circular structure termed a “β-propeller” to facilitate scaffolding through multiple protein: protein interactions [57]. The impact of the Leu307Arg substitution could be modeled on the available X-ray crystal structure of the Gβ5L:RGS9 complex (Figure 1E). Leu307 lies within a hydrophobic groove on the superior surface of the β-propeller of Gβ5L. This substitution would affect the electrostatic surface potential in the groove (substitution of a bulky hydrophobic residue with a long, positively charged side chain), and significantly alter local protein structure. In the Gβ5L:RGS9 complex, an amphipathic (but predominantly hydrophobic) helix from RGS9 (residues 239–255) lies directly in the Gβ5L groove, providing a large binding surface. The solvent accessible area excluded by the helix binding is 787 Å^2^, 19% of the total binding surface area in the heterodimer. Substitution of Leu307Arg would affect the Gβ5L:RGS9 interaction, pathologically altering quaternary structure and signaling complex formation. Our model of Gβ5L consists of residues 51–395, consistent with the known ordered structure for homologous proteins (PDB ID 2PBI). The only variance between Gβ5L and Gβ5S is that the latter does not possess residues 1–42, which likely forms a disordered N-terminal tail in Gβ5L and does not appear in our model or have predicted interaction with RGS proteins. Therefore, the model presented would apply equally to Gβ5L and Gβ5S.

## 4. Discussion

As a recently discovered genetic syndrome, the phenotypic spectrum of *GNB5*-related disorders is yet to be fully uncovered. Previously, most biallelic missense variants in *GNB5* have only been described in patients with normal intellect to mild ID with or without cardiac involvement, more in keeping with LADCI [12,13]. Here, we report a patient with a homozygous missense variant in *GNB5* who had severe ID, retinal electrophysiological changes, and abnormal cardiac rhythm more in keeping with IDDCA. However, the contribution of the *GNB5* variant to proband’s ID was complicated by the presence of 3-MCC deficiency, also previously thought to cause mild ID [58,59]. Recent literature on 3-MCC deficiency suggests very low disease penetrance and propose that the neurological symptoms may be unrelated to the disorder [36,60]. Hence, the severe ID in the proband is most consistent with *GNB5*-related disorder; however, further investigation is required to examine for any interactions between the two molecular diagnoses. The asystole and sinus bradycardia found in the proband is consistent with *GNB5*-related disorder. Previously, we characterized a dual retinal signaling defect in a patient harboring homozygous nonsense variant affecting both transcripts of GNB5 [15,33]. The identified defect is unique and affected both photo-transduction recovery (bradyopsia) and rod ON-bipolar signaling; bradyopsia was related to defective Gβ5L function in both rod and cone photoreceptors, whereas rod ON-bipolar signaling defect was related to defective Gβ5S function. The proband in the current study showed a selective defect in cone photo-transduction recovery, suggestive of defective Gβ5L function in cones; Gβ5L function in rods and Gβ5S function in rod ON-bipolar cells appeared normal. Since both rod and cone photoreceptors expressed Gβ5L, the sparing of rod photo-transduction recovery in the proband was unusual, but a similar instance has been previously reported with *R9AP*-related bradyopsia [61]. The proband was also diagnosed to have HbE trait following an incidental finding of splenomegaly. This case is an excellent example that highlights the importance of a multidisciplinary clinical and research approach incorporating genotype-specific reverse phenotyping and functional assays to aid variant classification in order to establish accurate clinical diagnosis in complex patients with overlapping traits.

Large WES cohort studies report 5–8% of patients as having multiple genetic diagnosis; this increases to 22% in consanguineous pedigrees [4,62]. In a patient cohort with an intellectual developmental disorder and metabolic phenotypes, 12% had coexisting monogenic conditions that contributed to the disease phenotype [63]. The distinction between an expanded phenotype versus comorbidity can be difficult with phenotypes that are common in Mendelian genetic disorders or in a particular population, such as neurodevelopmental disorders and myopia [4,64]. This distinction is further complicated when the phenotypic spectrum of the condition is not fully understood. In the present study, the suspicion for a second diagnosis was raised when the neurodevelopmental phenotype was found to be out of keeping with 3-MCC deficiency. Such clinical intuition is only possible when routine patient follow-ups are arranged, and detailed assessment is obtained at each follow-up.

The *in vitro* evidence from the BRET assay was crucial to the investigation of the functional impact of the missense variant. The findings indicate that the Leu265Arg substitution compromised the stability of Gβ5S protein, which in turn failed to stabilize GAP complex containing catalytic RGS9-2 subunit. Thus, the variant generated a strong loss-of-function phenotype. Since Gβ5L isoform is exclusively expressed by photoreceptors, functional study of the Gβ5L mutant variant would ideally be carried out in photoreceptor cell lines; nevertheless, the ERG findings in the proband are consistent with defective Gβ5L function in cone photoreceptors. Overall, BRET assay may be used for drug screening in rare genetic disorders such as *GNB5*, where personalized therapeutics may be the only avenue [65]. Structural analysis indicates that the mutation is located within the conserved WD40 repeat domain of Gβ5, which is directly involved in the protein–protein interaction with RGS9.

## 5. Conclusions

On the basis of the proband’s phenotype and in vitro functional assays, we have reclassified the *GNB5* missense variant (*GNB5L*: (p. Leu307Arg); *GNB5S*: (p. Leu265Arg)) as likely pathogenic as per ACMG variant classification criteria (PS3, PM2, PP3). The cardiac and neurodevelopmental phenotype is consistent with defective Gβ5S function. The ERG phenotype is suggestive of defective Gβ5L function in cones. This study highlights the need for precise phenotyping and functional assays to aid variant classification in complex disorders. Further, this study emphasizes the importance of continued follow-up in patients when the phenotype is not consistent with the genotype, in order to explore the possibility of additional genetic diagnoses. 

## Figures and Tables

**Table 1:** Comparison of the phenotypic features between 3-methylcrotonyl-CoA carboxylase (3-MCC) deficiency, *GNB5*-related disorders, hemoglobin E trait, and that seen in the proband.

	3-MCC	GNB5 IDDCA; MIM: 617173	GNB5 LADCI; MIM: 617182	Hemoglobin E Trait	Proband
Neurology	Seizures Lethargy Hypotonia	Epilepsy Encephalopathy Hypotonia	Hypotonia	None	Febrile seizure Encephalopathy Central hypotonia
Neurodevelopmental	Developmental delay Psychomotor retardation Mental retardation Not always present	Delayed psychomotor development Intellectual disability (severe) Speech dela	Speech delay Intellectual disability (mild) ADHD	None	Global developmental delay Intellectual disability (severe) None verbal
Ophthalmology	None	Nystagmus Abnormal ERG: - Bradyopsia - Rod ON-bipolar - dysfunction	None	None	High myopia ↓flicker amplitude Subtle cone photo-transduction recovery deficit
Cardiovascular	None	Sick sinus syndrome Sinus bradycardia Escape beats	Sick sinus syndrome Bradycardia Arrhythmias (in some patients)		Asystole Sinus bradycardia Escape beats
GI	Liver: steatosis Feeding difficulty	Pathological gastric reflux	None	None	Poor feeding Sialorrhea
Hematology	None	None		Anemia Splenomegaly	Mild splenomegaly Transient increase in hemoglobin
Respiratory	Apnea	None	None	None	Obstructive sleep apnea
Metabolic	Episodic metabolic acidosis, Metabolic decompensation precipitated by illness Hypoglycemia 3-methylcrotonyl glycine on UOA	(One patient with increased urine 3-methyl-glutaconic acid)	None	None	3-Methylcrotonylglycine on UOA
Constitutional	Failure to thrive	None	None	None	None
